# Minor lobulation of the testis, mimicking polyorchidism when inflammed, discussion of a rare case: A case report

**DOI:** 10.1016/j.ijscr.2022.107448

**Published:** 2022-07-23

**Authors:** Mohammad Hadi Gharib, Reza Zahedpasha

**Affiliations:** Department of Radiology, School of Medicine, 5th Azar Hospital, Gorgan, Golestan, Iran; Golestan University of Medical Sciences, Gorgan, Golestan, Iran

**Keywords:** Case-report, Bilobed testis, Polyorchidism, Ultrasound, MRI

## Abstract

**Introduction:**

*Bilobed testis* is an uncommon congenital malformation with only eight cases reported up to now. It seems that bilobed testicle is a form of polyorchidism which is not yet thoroughly divided.

This report could provide information about diagnosing minor lobulation on ultrasound and MRI for the first time.

**Presentation of case:**

In this report, a 13-year-old boy presented with extreme Epididymo-orchitis on the right testis, without any history, which showed itself on ultrasound as type A3 polyorchidism or bilobed testis.

**Clinical discussion:**

Recent studies have not shown an apparent association between bilobed testis with testicular torsion and malignancy. In our case, because the minor lobulation is small, it probably has no association with torsion. The bilobed testis seems benign, so there is no requirement to check tumor markers. An inflamed testicular appendix and epididymitis can appear similar to a major lobulation which must be accurately found and evaluated on ultrasound as separate entities.

**Conclusion:**

Inflamed minor lobulation of the testicle can demonstrate itself as polyorchidism or bilobed testicles; thus, Ultrasound and MRI can assist in diagnosing minor lobulation. Serial examination and imaging are recommended for managing minor lobulation.

## Highlights

• How to detect bilobed testis in ultrasound
• MRI was used for the first time to diagnose minor lobulation of the testis.
• The bilobed testis seems benign, so there is no requirement to check tumor markers.
• There is probably no association between minor lobulation of testicles and testicular torsion.
• Bilobed testicle can be evaluated by serial examination and imaging.

## 1 Background

Bilobed testis is a rare congenital anomaly that has been reported in less than ten cases to date [1]. The etiology of the bilobed testis is speculated to be an incomplete division of the genital ridge secondary to a peritoneal band [2]. The bilobed testis is assumed to be a form of polyorchidism [3]. The conclusion is that the bilobed testicle has always been a benign condition due to recent investigations [2]. Inguinal hernia, undescended testes, testicular torsion, and also the risk of malignancy have been related to polyorchidism [4]. Ultrasound and MRI generally help diagnose the bilobed testicle, polyorchidism and rule out malignancy [5].

In this case study, a 14-year-old boy with minor lobulation in the right testis was diagnosed by MRI, similar to polyorchidism in previous ultrasounds when he had Epididymo-orchitis.

## 2 Case presentation

**Fig. 1:**
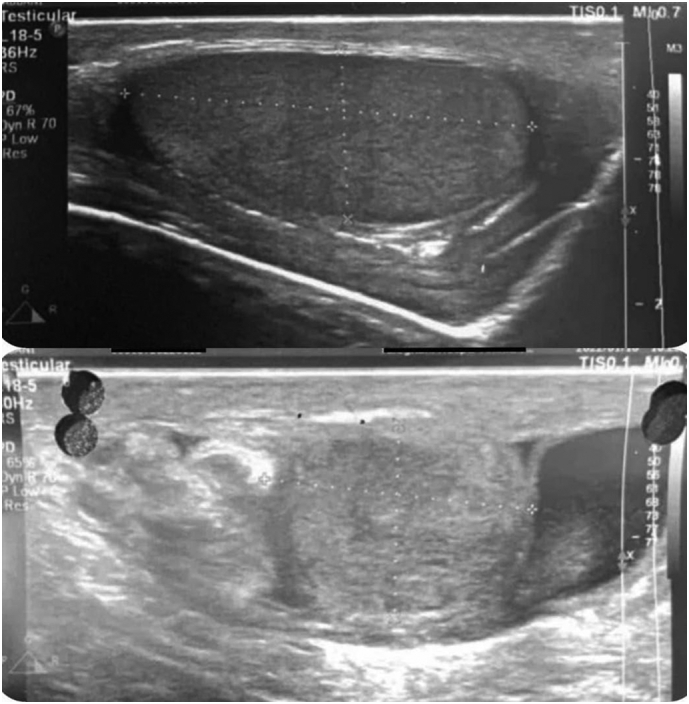
Ultrasonography. Sonography at acute phase demonstrating normal size right testis (a) and small testicular structure with similar background echotexture to the right testis, located superior to right testis; edematous and enlarged at the time of study due to inflammatory process (b).

A 13-year-old boy was referred to Taleghani Children's Hospital with a complaint of swelling and pain in his Right lower quadrant and right testicle, erythema, and warmth on the scrotum without any Past medical history, drug history, and surgical history who had fallen to the ground three days ago. Ultrasound results revealed that the right testicular appendix is prominent, but it also has a hypoechoic structure that resembles the testicular parenchyma. This structure is above the right testicle and the medial part of the right epididymis, is wholly attached to the testicle, and has 17 × 12 mm dimensions. Furthermore, in color Doppler, the coloration is sharply increased (Fig. 1).

The above findings suggest extreme Epididymo-orchitis on the right and maybe a type A3 polyorchidism with adjacent extra testis or a bilobed testis.

**Fig. 2:**
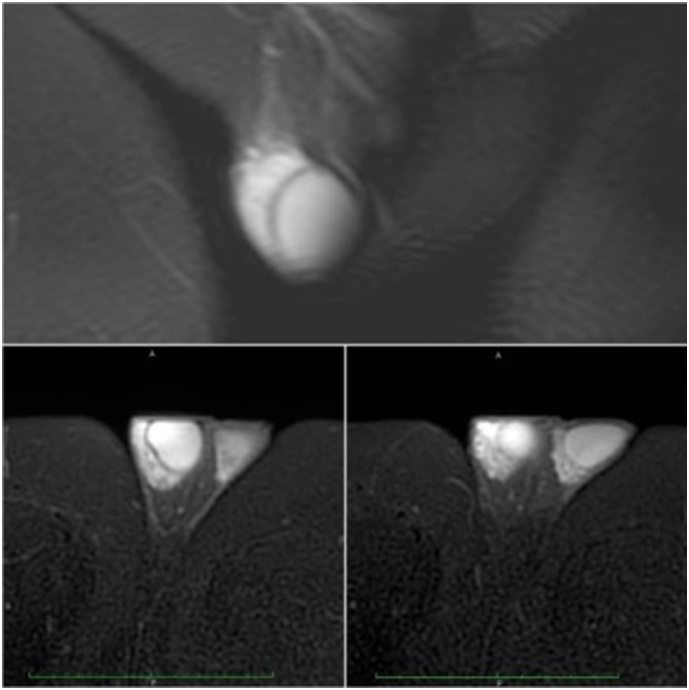
MRI. MRI after completion of antibiotic therapy for further evaluation of the proposed diagnosis in acute phase by sonography; In axial and coronal T2 FISP images, hemitesticular structure with similar signal intensity to that of the adjacent normal right testis is seen, now decreased in size due to resolution of inflammatory phase.

After treatment of the Epididymo-orchitis, Magnetic resonance imaging (MRI) was performed one month later. In axial and coronal T2 FISP images, a Hemi-testicular structure of similar signal intensity to that of the adjacent normal testis is noted on the right side, albeit with significantly decreased size due to the resolution of the inflammatory phase (Fig. 2).

## 3 Discussion

It is essential to differentiate the bilobed testis from polyorchidism because polyarchidism can be associated with testicular torsion and malignancy [4]. However, previous reports have shown no direct link between testicular torsion and malignancy with the bilobed testis. One of the differences between bilobed testis and polyorchidism is that on ultrasound of polyarchidism, the extra testicle is smaller than the main testicle [6]. Nevertheless, a deep horizontal gap in the bilobed testis is observed in the testicle with the same echogenicity [6]. In our case, because the minor lobulation has a small size, the risk of torsion can be even lower than that of bilobed testis with larger lobulations and polyarchidism, and even conclude that it probably has no association with torsion.

Bilobed testis appears to be a type of polyorchidism. There is a 5.7–7 % chance of malignancy in polyorchidism cases, which is higher than the usual risk of the general population,0006 % [3]; however, there has been no evidence that bilobed testis is associated with malignancy, and therefore, the tumor markers like alpha-fetoprotein (AFP)and beta-human chorionic gonadotropin (Beta-hCG) is not necessary to be measured in these cases [1], [2]. Thus, bilobed testis seems benign [2]; Therefore, serial examination and imaging are preferable to surgical management [1], [2]. Few studies have been done on the bilobed testis because the number of bilobed testes is much less than in polyorchidism. For this reason, we perform the procedures mentioned in the studies, including serial examination and imaging.

There is a greater tendency for infection in minor lobulation due to problems in the drainage of sperm or blood supply. Due to infection and subsequent inflammation, this minor lobulation demonstrates more pronounced swelling and inflammation than the main part of the testicle and hence may seem to be polyorchidism or a major lobulation; the testicular appendix and epididymis can also become inflamed and appear similar to major lobulation.

The appendix and testicular epididymis must be accurately found and evaluated on ultrasound as separate entities. After that, if there is another element mimicking the parenchymal structure of the testicle on ultrasound, we should think of minor lobulation that is inflamed and swollen. In these situations, minor lobulation shows itself similar to major lobulation.

## 4 Conclusion

Polyorchidism is commonly associated with complications such as testicular torsion and malignancy, while there is probably no association with torsion in minor lobulation of the testis; therefore, distinguishing bilobed testis from polyorchidism through ultrasound and MRI can be beneficial for us.

Since Minor lobulation of the testis seems to be benign malformation and the possibility of torsion and other complications are low, serial examinations and imaging are recommended for managing these cases.

## 5 Methods

This case report has been reported in line with the SCARE Criteria [7] at the end of the introductory section.

## Patient perspective

The patient and the patient's family were satisfied with all the work that was done for them, and the patient's family said, “Thank you for the work you have done to find out about our child's illness.”

## Funding

There was no funding.

## Ethical approval

It is not required.

## Consent

Written informed consent was obtained from the parents for publication of this case report and accompanying images. A copy of the written consent in our native language is available for review by the Editor-in-Chief of this journal on request.

## Author contribution

None.

## Registration of research studies

None.

## Guarantor

The corresponding author is the guarantor of submission.

## Declaration of competing interest

There is no relationship.
